# Suicide and suicidality in Australian Defence Force veterans: A systematic scoping review

**DOI:** 10.1177/00048674241246443

**Published:** 2024-04-22

**Authors:** Csongor G Oltvolgyi, Carla Meurk, Ed Heffernan

**Affiliations:** 1 School of Public Health, The University of Queensland, Herston, QLD, Australia; 2 Queensland Centre for Mental Health Research, Wacol, QLD, Australia; 3 Queensland Forensic Mental Health Service, Brisbane, QLD, Australia

**Keywords:** Suicide, suicidality, veteran, military

## Abstract

**Objective:**

Increased suicidality and suicide deaths among veterans of the Australian Defence Force have gained recent prominence. A systematic scoping review was conducted to identify, summarise and synthesise the existing literature relating to Australian veteran suicide and suicidality, with the objective of identifying future research priorities.

**Methods:**

We conducted a PRISMA-compliant systematic search on PubMed/MEDLINE, Embase and CINAHL databases for all manuscripts reporting primary data on suicide and suicidality in Australian veterans. The search was supplemented by grey literature and a search of reference lists. Manuscripts of any study type, published in the English language since the Vietnam era, were eligible for inclusion.

**Results:**

A total of 26 articles and reports, utilising a variety of mostly quantitative approaches, were included in the review. Findings, especially in larger and more recent studies, indicate increased suicidality in the veteran population. Suicide deaths appeared to increase with transition out of the military. Mental illness was identified as an important risk factor for suicide and suicidality. Current service was identified as a protective factor against suicide. There was mixed evidence regarding the impact of operational deployment on suicide and suicidality.

**Conclusions:**

Gaps were identified in relation to the relative contributions to risk from transition, the various psychosocial correlates (for example, relationships, finances, employment), pre-service factors and the extent to which these are causal or mediating in nature. A better understanding of health service utilisation would also aid in targeting preventive efforts. Future research in these areas is warranted.

## Introduction

Suicide deaths among ex-serving members of the Australian Defence Force (ADF) are significantly higher than that of the wider Australian community (AIHW, 2022). Similarly, the incidence of mental illness and suicidality are also significantly elevated in ex-serving personnel (McFarlane et al., 2011; Van Hooff et al., 2018). Accordingly, commencing in 2021, the Royal Commission into Defence and Veteran Suicide (the ‘Royal Commission’) has been established to investigate suicide, suicidality and mental ill health in current and ex-serving members of the ADF. The Letters Patent establishing the Royal Commission recognise ‘the unique nature of military service, and the ongoing impact such service may have on the physical and mental health of defence members and veterans’ and that ‘there is an overrepresentation of defence and veteran deaths by suicide in Australia, and that this overrepresentation should be acknowledged and understood to ensure that learnings are made and to prevent future deaths by suicide’ (Commonwealth of Australia, 2022). This scoping review seeks to support this goal by synthesising existing evidence and identifying gaps in knowledge regarding veteran suicide and suicidality.

Military service is a unique occupation. The ADF is a small but highly professional force of officers, sailors, soldiers and aviators who choose a career of service in defence of Australia and its national interests and, in doing so, forgo some of their liberties and accept a degree of the risk to their health, and indeed life, that such service may bring about. The uniqueness of this occupation translates into specific differences in how health care, including mental health care, is delivered and accessed. Important cultural considerations when assessing the mental health of current and ex-serving members of the ADF have been outlined by Lane and Wallace (2020), and include such factors as military norms, values, identity and purpose.

### Veteran population

The definition of a veteran has evolved over time and can be confusing. Traditionally associated with having returned from active operational service (for example, war or peacekeeping), a meeting of Australian Veterans’ Ministers agreed in 2017 to adopt a cross-jurisdictional definition of veteran to include anyone who has served at least one day in the ADF (Veterans’ Ministers Roundtable, 2017). Therefore, the definition of veteran now includes currently serving and ex-serving people. The Royal Commission further delineates a current serving ADF member as ‘any person currently serving as a member of the Australian Defence Force, whether permanent forces or reserves, and who has served at least one day’ and an ex-serving member as ‘any person who has served in the Australian Defence Force, whether permanent forces or reserves, and who served at least one day and has since discharged from the Australian Defence Force’ (Commonwealth of Australia, 2022). This review adopts the abovementioned definitions, with exceptions to this made explicit where different specific definitions are used in the included studies. Box 1 outlines the respective population sizes of current and ex-serving members of the ADF.

**Box 1.** Current and ex-serving members of the ADF.

• There are approximately 59,000 currently serving members in the ADF permanent force and 38,700 in the reserves (AIHW, 2021). • With the introduction of a new question regarding lifetime military service in the 2021 Australian Census, it is now possible to estimate the number of ex-serving members in Australia. • The 2021 census estimated that there are approximately 500,000 ex-serving members in Australia (Australian Bureau of Statistics, 2021).

### Suicide and suicidality

Measuring suicide and suicidality is a complex endeavour. Suicide incidence has widely been considered to be systematically underestimated due to limitations in being able to establish intent post-mortem, and because suicide is often only identified as such after the exclusion of other potential causes of death (Bertolote and Wasserman, 2021). Conversely, coding of suicide in Australia includes instances where a person has harmed themselves intentionally, without fatal intent, but consequently died (Biddle et al., 2020). Thus, there are measurement biases which may contribute to either over- or underestimating the true rate of suicide. In Australia, the establishment of cause of death when it is unexpected rests with the coroners of each jurisdiction (Kerridge et al., 2013). There is no legislated definition of suicide in Australia, and coronial discretion varies across jurisdictions (Jowett et al., 2019). Furthermore, the coronial process takes time and can be subject to revision (Biddle et al., 2020), and has no consistent way of identifying occupation or military status of the deceased (Commonwealth of Australia, 2022). Thus, identifying suicide deaths of veterans can only be established by data linkage between Australian death register data and Defence records (AIHW, 2021).^1^

Suicidality is no less difficult to define and there are no universally agreed definitions (Favril et al., 2023; Kapur and Goldney, 2019). Suicidality is a heterogeneous grouping and can include suicidal ideation, suicidal thoughts of varying intensity, active plans, suicidal gestures, behaviours or attempts (which in some cases will include self-harm without fatal intent) and in some definitions suicide as well (Bertolote and Wasserman, 2021). Many instances of suicidality will not be reported and therefore not captured by research or in health records or relevant registers. In this review, we use the term suicidality to mean suicidal ideation, suicidal thoughts of varying intensity, active plans, suicidal gestures, behaviours or attempts.

### The link between suicidality and suicide

Risk factors for suicide and suicidality are numerous and complex (Turecki et al., 2019). It is recognised that mental ill health is an important risk factor for both suicidality and suicide death (Harris and Barraclough, 1997), and interventions for mental ill health can be a significant point of intervention. There are also a variety of theoretical models that seek to explain the necessary components, steps or motivations that result in suicide death (Joiner, 2005; Klonsky and May, 2015; O’Connor, 2011), including sociological and/or cultural drivers, access to means and development of capacity to move from suicidality to suicide.

This scoping review aims to identify, summarise and synthesise the current literature landscape in Australian veteran suicide and suicidality, and then use the findings to identify priorities for future research.

## Methods

A scoping review was determined to be the most appropriate approach to achieving the stated aim (Munn et al., 2018; Peters et al., 2020). The scoping review methodology and reporting was guided by the Preferred Reporting Items for Systematic Reviews and Meta-Analyses extension for Scoping Reviews (PRISMA-ScR) 2018 guideline (Tricco et al., 2018). The review protocol was registered a priori with Open Science Framework (https://doi.org/10.17605/OSF.IO/R5ZEK). Ethical approval was not required for this scoping review of published peer-reviewed literature and reports.

### Information sources

On 23 January 2023, a systematic search was conducted on PubMed/MEDLINE, Embase and CINAHL. There was no restriction on publication type, so long as findings based on primary data collection were reported. Search results were supplemented by grey literature relevant to the area (predominantly government reports and unpublished research), and reference lists of eligible manuscripts were searched for additional articles of relevance.

Search terms were formulated with the assistance of a research librarian at the University of Queensland. The search strategy included <suicide/suicidality terms> AND <military/veteran terms> AND Australia. The full details of the search strategy are available at Supplementary Materials 1.

### Eligibility criteria

The population of interest in this scoping review is Australian veterans, using the broad definition adopted by the Royal Commission (including both current and ex-serving, and those who have deployed on operations and those who have not). The phenomena of interest were suicide and suicidality. Research reporting on thoughts and behaviours along the spectrum from ideation to completed suicide were eligible for inclusion. Research from all settings, including in-patient, out-patient and community samples (whether or not accessing health care), were considered for inclusion. Only research relating to people serving during the Vietnam era and onwards were included. Articles reporting primary data were included regardless of study type and they were restricted to publications in the English language (not considered a limitation for the population of Australian current and ex-serving military).

### Data screening and extraction

Duplicates returned in the search strategy were removed using the Covidence web-based collaboration software platform (Veritas Health Innovation, 2023). Screening of search results was then conducted by C.G.O. across two phases: title/abstract and full text. Screening was cross-checked by C.M. and conflicts resolved by a third screener, if necessary. After screening, data were extracted by C.G.O. and reviewed by C.M. The research team developed a data extraction form to include citation details, participant characteristics, sample size, study design, funding source, construct measured (suicide/suicidality and associated definition), tools used (psychometrics, coroner database, etc.) and relevant outcomes.

### Data analysis and synthesis

Consistent with the purpose of a scoping review, a qualitative descriptive synthesis of included articles and reports was performed. The characteristics of the included materials were tabulated and key themes analysed and summarised.

## Results

### Study characteristics

**Figure 1.**
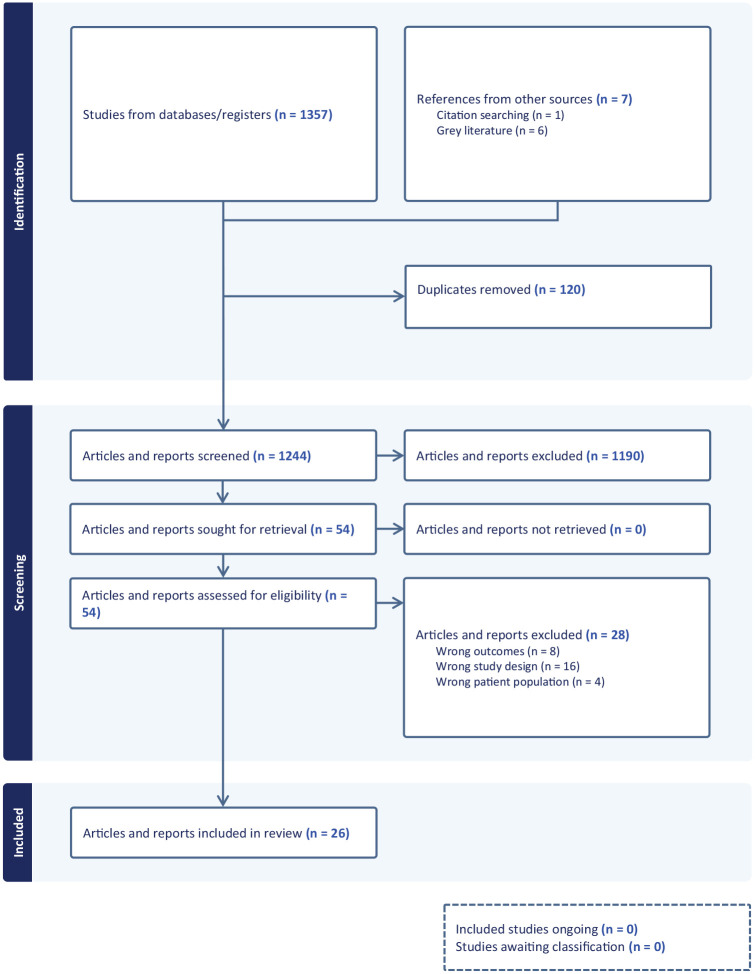
PRISMA flow diagram.

Figure 1 depicts the PRISMA flow diagram (Page et al., 2021). Where government reports had multiple iterations and updates, only the most recent update was included in the review. Table 1 lists the 26 articles and reports which were included in the review.

**Table 1.** Included articles and reports.

Citation	Participants	Sample size	Years of study	Aims	Study design	Outcomes measured; definitions	Suicide/case ascertainment
Duncan et al., 1974	Current serving Permanent Army	194 members identified as having self-inflicted injury across four defence-wide databases	1970–1972	To examine incidence of suicide, attempted suicide and suicidal gestures	Retrospective audit	Suicide; self-harm behaviours Substances used in overdose Psychiatric diagnoses	Reporting by Central Army Records, Royal Australian Army Provost Corps, medical and psychology units
O’Toole et al., 2015	Ex-serving Army Vietnam veterans	448 respondents out of random sample of 1000 taken from Army-supplied roll	2005–2006	To examine relative risk of suicidality and the influence of mental illness	Cross-sectional	Suicidality Psychiatric diagnoses	Report in structured interview
Fett et al., 1987b	All National Servicemen (conscripts) Vietnam veteran or era	19,205 Vietnam veterans 25,677 Vietnam era	1966–1982	To examine causes of death among Vietnam veterans	Retrospective cohort	Cause of death Suicide deaths	Independent assessment by five physicians of death certificates and available records from hospitals, doctors, coroners, police, the Army, courts and governments
Forcier et al., 1987	Vietnam veterans (conscripts)	19,205 Vietnam veterans (same cohort as Fett et al., 1987b)	1966–1982	To assess impact of location or time of deployment to Vietnam on suicide risk	Retrospective cohort	Deaths (including suicide deaths) Examined by time and location of service	Medical, hospital and coroner records
Boman, 1985	Vietnam veterans (not further defined) All inpatients at Sydney Repatriation Hospital, 1981–1984	50 veterans (21 with PTSD, 27 without)	1981–1984	To examine association of PTSD with suicide attempt in Vietnam veterans	Case series	Psychiatric comorbidity Suicide attempts	Report in clinical interview
Adena et al., 1985	All National Servicemen (conscripts) Vietnam veteran or era	19,205 Vietnam veterans 25,677 Vietnam era (same cohort as Fett et al., 1987b)	1966–1982	To examine relative incidence of suicide between those conscripts who deployed to Vietnam and those who did not	Retrospective cohort	Cause of death	Medical, hospital and coroner records
O’Toole and Cantor, 1995	All National Servicemen (conscripts)	19,430 Vietnam veterans 27,081 Vietnam era	1966–1982	To examine risk factors for suicide among Vietnam conscripts	Case control	N/A–case control Cases were suicide deaths	Medical, hospital and coroner records
O’Toole et al., 1988	All National Servicemen (conscripts)	Not stated, but cohort as Fett et al., 1987b	1966–1982	To examine association of psychiatric conditions with all-cause mortality, which included a known number of suicides	Case control	N/A–case control Cases were all deaths	Medical, hospital and coroner records
O’Toole et al., 2010	Male Army Vietnam veterans	1000 male Army Vietnam veterans	Up to 2004	To measure suicide incidence in a cohort of Vietnam veterans	Prospective cohort	Deaths	State and Territory death registries
Kerr et al., 2018	Ex-serving PTSD day programme patients	229 patients	2007–2014	To examine correlates of suicide attempts by veterans	Retrospective cohort	Suicide attempt (self-report)	Suicide attempt self-report
Kerr et al., 2021	Ex-serving PTSD day programme patients	219 patients	Unclear	To examine association between guilt and suicide attempt	Retrospective cohort	Suicide attempt (self-report)	Suicide attempt self-report
Bryant, 1998	Callers to Vietnam Veterans’ Telephone Counselling Service	274 calls	1990s–unclear exact year	To examine reasons for calls to counselling service	Cross-sectional	Suicide risk	Unstructured
McLaughlin and Hamilton, 2019	Users of a veteran support organisation who have PTSD and have been paired with a service dog	7 volunteers	2010s–unclear exact year	To examine protective effects of service/assistance dogs	Semi-structured focus groups	Qualitative–suicidality, suicide averted	Self report
Theal et al., 2020	Vietnam veterans with PTSD Recruited from participating hospital, online and print advertising and word of mouth	160	2014–2015	To examine association between psychotropic polypharmacy and suicidality	Cross-sectional	Suicidality using structured interview	Structured interview–MINI
Varker et al., 2022	TWRP sample	12,806 current and ex-serving	2015–2016	To examine association of problem anger with suicidality	Cross-sectional	Suicidality	Structured interview
Forbes et al., 2016	Random selection from roll of peace keepers provided by Defence and DVA	1025 participants	Unclear	To examine incidence of suicidality among former peace keepers	Cross-sectional	Suicidality Psychiatric diagnoses Potentially traumatic events	Self-report of suicide ideation, plan and attempt
Milner et al., 2017	Emergency and protective service workers NCIS deaths	All suicides in NCIS	2001–2012	To examine relative risk of suicide in (current serving) Defence compared with other protective service occupations	Retrospective case-series	Suicide deaths	NCIS ‘intentional self-harm’
Gisler and Sadler, 2000	Current serving ADF Unclear if any Reserves	Whole ADF	1985–2000	To catalogue ADF suicide	Case series	Suicides Methods of suicide	Not described
Syed Sheriff et al., 2019	All ADF and matched civilians	MHPW sample	2010–2011	To examine relationship between childhood trauma and suicidality, and compare this with matched civilians	Cross-sectional	Suicidality Mental disorder	Structured interview
Syed Sheriff et al., 2020	Sample of transitioned 2010–2014	TWRP sample	2015–2016	To examine relationship between childhood trauma and suicidality in recently transitioned veterans	Cross-sectional	Suicidality Mental disorder	Structured interview
McFarlane et al., 2011 **(MHPW)**	All permanent current serving in ADF	24,481 respondents (of 50,049 serving)	2010	To measure incidence and assess correlates of suicidality in serving members of ADF	Cross-sectional	Suicidality (self-report phase) Mental disorder	4 questions re suicidality CIDI for diagnostic purposes
Van Hooff et al., 2018 **(TWRP).** **(Includes component reports** Bryant et al. (2019) **and** Lawrence-Wood et al. (2019))	Multiple cohorts: - permanent serving - reservist - family members - longitudinal from MHPW	12,806 permanent ADF 1163 reserve 8497 longitudinal from MHPW	2015	To measure incidence and assess correlates of suicidality in serving members of ADF and those recently transitioned	Mixed: Cross-sectional Cohort (from MHPW)	Suicidality (self-report phase) Mental disorder	4 questions re suicidality CIDI for diagnostic purposes
AIHW, 2021	216,640 living and deceased ADF members (service since 2001)	All suicides (465)	2001–2018	To measure total suicides and correlates in cohort of veterans	Cohort–linkage	Suicide deaths Multiple risk and protective factors MBS and PBS data	NCIS
AIHW, 2022	379,000 living and deceased ADF members (service since 1985)	All suicides (1600)	1997–2020	To measure total suicides and correlates in cohort of veterans	Cohort–linkage	Suicide deaths Multiple risk and protective factors	NCIS
Sim et al., 2015	All veterans of 1991 Gulf War Comparator from 2003	715 Gulf War veterans 675 comparison	2011–2013	To assess association of Gulf War service with suicidality	Cohort	Suicidality	Same 4 questions as MHPW
Wilson et al., 2005	All Australian personnel who served in Vietnam 1962–1973	59,179 male veterans	Up to 2001	To assess suicide mortality in deployed Vietnam war veterans and compare with general Australian population	Retrospective cohort	Suicide deaths	NDI

ADF: Australian Defence Force; DVA: Department of Veterans’ Affairs; PTSD: Posttraumatic Stress Disorder; NCIS: National Coronial Information System; NDI: National Death Index; MHPW: Mental Health Prevalence and Wellbeing study; TWRP: Transition and Wellbeing Research Programme; CIDI: Composite International Diagnostic Interview; MBS: Medicare Benefits Schedule; PBS: Pharmaceutical Benefits Schedule.

Eleven articles and one government report, with publication dates ranging from 1985 to 2020 examined the experience of veterans of the Vietnam conflict and era. The remainder were either not associated with any particular conflict or examined veterans of more recent conflicts or peacekeeping operations. The most common research designs were cohort (*n* = 10) and cross-sectional (*n* = 9), although case-control (*n* = 2), case series (*n* = 3), qualitative approaches (*n* = 1) and mixed methods (*n* = 1) were also identified.

### Suicide^2^

A small retrospective audit reported seven suicide deaths among full-time serving members of the Australian Army between 1970 and 1972 (Duncan et al., 1974). While not adjusted for age, the incidence of suicide deaths was calculated to be lower than the Australian community figure of 13.6/100,000/year.

A large retrospective cohort study was commissioned by the Commonwealth Government of Australia in 1981 (the Australian Veterans’ Health Studies Mortality Study) in order to investigate whether service in the Vietnam conflict was associated with premature all-cause and/or specific mortality. The cohort consisted of National Servicemen (conscripts) who enlisted in the Australian Army during the Vietnam conflict and served at least 12 months; 19,205 of these men served in Vietnam and 25,677 had service limited to Australia. Suicide accounted for 15% of the 523 deaths recorded in this cohort between 1966 and 1982 (Adena et al., 1985). The all-cause mortality for the whole cohort between 1966 and 1982 was lower than age-matched Australian males, although higher in the group that served in Vietnam compared with those who did not (Fett et al., 1987a). A subsequent paper reported 82 suicide deaths among the cohort between 1966 and 1982 (Fett et al., 1987b). While no comparison was made to community suicide rates, the suicide rate was 1.5 times higher among those who served in Vietnam than those who did not. This relative risk reduced to 1.3 and lost statistical significance when rates were adjusted for corps of service, the Royal Australian Engineers accounting for the excess deaths. This was considered due to higher suicide mortality in corps with greater proportion of conscripts who were deployed to Vietnam (Fett et al., 1987b). There was no significant effect of time or location of deployment in Vietnam (Forcier et al., 1987). The overall conclusion was of ‘qualified support’ for stress-related disorders being over-represented in veterans of the Vietnam conflict (Fett et al., 1987b).

A prospective cohort study of a random sample of 1000 Australian Vietnam veterans reported 117 deaths up to December 2004, of which 13 (11.1%) were suicide deaths (O’Toole et al., 2010). While this study did not detect associations between combat exposure or psychiatric diagnoses and all-cause death, the relationship between these variables and suicide deaths specifically was not reported.

Subsequent suicide statistics on serving ADF members were published in internal technical reports (Bounty et al., 2004 as cited by Sadler et al., 2021), although there is one short published report of findings from 1985 to 2000 (Gisler and Sadler, 2000). This report identified a total of 142 suicide deaths in current serving members from 1985 to 2000, equivalent to 14.3/100,000/year. This rate was similar to crude community rates at the time, although was less than rates for young Australian men. There is also a brief published report of suicide among various emergency and protective service workers, including current serving Defence personnel, based on Australian coronial data from 2001 to 2012 (Milner et al., 2017). This study showed an elevated age-adjusted suicide rate in the whole cohort (without providing a specific rate for the military sub-group only) compared with other occupations. An adjusted relative risk of suicide of 3.27 in the military group compared with all other occupations was the highest among the various emergency and protective service workers reported in the study (the others being ambulance, police, firefighters and prison/security officers). There was no comparison with the general community.

More contemporary Australian military suicide statistics are found in the grey literature. The Australian Institute of Health and Welfare (AIHW) has released an annual report on serving and ex-serving suicide deaths since 2017, utilising linked data from Defence personnel systems and the National Mortality Database (NMD). An additional report, based on data up to the end of 2018, was released in 2021 (AIHW, 2021) to support the terms of reference of the Preliminary Interim Report of the Interim National Commissioner for Defence and Veteran Suicide Prevention (Commonwealth of Australia, 2021). This report stated that 465 serving and ex-serving members of the ADF had died by suicide between 2001 and 2018. The standardised mortality ratio (SMR) for serving and ex-serving males was 0.50 and 1.22, respectively, while for females it was 0.47 and 2.27,^3^ respectively (AIHW, 2021). The suicide rate was higher in general enlistees than officers, those with shorter periods of service and in those subject to involuntary separation on medical grounds. Associations with mental illness and psychosocial risk factors are further outlined below.

The most recent AIHW annual report was expanded to report on serving and ex-serving suicide deaths between 1997 and 2020, in members who had served since 1985 (AIHW, 2022). As at the end of 2020, almost 379,000 Australians had served at least one day in the ADF between 1985 and the end of 2020. Of these, approximately 263,000 were ex-serving, 60,000 were in the permanent forces and 39,000 were in the reserves. The total number of deaths by suicide between 1997 and 2020 was 1600. Of these, 1471 were male and 1330 were ex-serving at the time of death. Compared to the age-matched Australian population, suicide rates were 49% lower for males in the permanent force, 46% lower for males in the reserves and 27% higher for ex-serving males. For ex-serving females, the suicide rate was 107% higher than the Australian community, although was lower in comparison to ex-serving males. Once again, the suicide rate in males subject to involuntary medical separation was significantly elevated at 69.8/100,000/year, compared with 12.6/100,000/year for serving males.^4^

### Suicidality

Duncan et al. (1974) reported 187 episodes of non-fatal suicide behaviour among full-time serving members of the Australian Army between 1970 and 1972, most of which they termed suicide ‘gestures’ (and which they define similarly to what is now usually referred to as non-suicidal self-injury, that is, absent fatal intent). They did not measure suicide ideation or planning and so likely under-report suicidality by a significant degree.^5^

Boman (1985) described a case series of 50 Vietnam veterans (all ex-serving) admitted to the Repatriation General Hospital in Sydney between 1981 and 1984. The author found no difference in the frequency of suicide attempts between those exposed to combat and diagnosed with post-traumatic stress disorder (PTSD), and those who were not.

An analysis of telephone calls made to a Vietnam veterans’ counselling service found that 10% of calls during a 9-week monitoring period were related to acute suicidality. Almost half of these were already engaged in formal counselling or therapy (Bryant, 1998).

The 2010 ADF Mental Health Prevalence and Wellbeing (MHPW) study (McFarlane et al., 2011) was the first comprehensive assessment of mental health in permanent members of the ADF, with 49% of the entire force participating in the study. Comparison was made with the Australian community by comparing with results of the 2007 National Survey of Mental Health and Wellbeing (Australian Bureau of Statistics, 2008), matched for age, sex and employment status. Overall, 4.0% of the ADF sample had reported any suicidality in the past 12 months, 3.9% having thought about attempting suicide and 1.1% having made a suicide plan. Each of these figures was more than double the matched Australian community. The 12-month incidence of reported suicide attempt was 0.4% in the ADF, which was similar to the community incidence of 0.3%. Females in the ADF were at higher risk of ideation than males, but not suicide attempts, similar to the matched Australian population.

In the later Transition and Wellbeing Research Programme (TWRP), suicidality in current serving members of the ADF was compared with members who had transitioned out of the permanent forces in the preceding 5 years (Van Hooff et al., 2018). This study detected significantly higher suicidality, including self-reported suicide attempts, in the transitioned cohort compared with the current serving cohort. Furthermore, 12-month self-report of any suicidality in serving members had increased from 4.0% in 2010 to 8.8% in 2015 (Van Hooff et al., 2019). In the 2015 transitioned cohort, there was no statistically significant difference in suicidality between those who had ever deployed on operations and those who had never deployed. There was a considerable increase in suicidality, including self-reported suicide attempts, in those members who had transitioned due to medical discharge rather than other reasons (Van Hooff et al., 2018).

Lifetime suicidality was assessed in a cohort of 448 ageing ex-serving Australian Vietnam veterans and compared with age- and sex-matched Australian population controls (O’Toole et al., 2015). Compared with the matched population, the lifetime relative risk of suicidal ideation in the Vietnam veteran cohort was 7.91; for suicide planning it was 9.73, and for self-reported suicide attempts it was 13.82. All of these were statistically significant results.

### Mental illness, suicide and suicidality

The relationship between a number of correlates of mental illness and suicide or suicidality has been assessed using linked data from Defence, the Department of Veterans’ Affairs (DVA) and Medicare (AIHW, 2021). Approximately 75% of male ADF members who had died by suicide had been diagnosed with at least one mental and behavioural disorder (defined as *International Classification of Diseases, Tenth Revision* [ICD-10] codes F00–F99), compared with 63% of age-matched Australian males who had died by suicide. The most common association was a depressive episode (diagnosed in 48.5% of ADF males who died by suicide), followed by alcohol-related disorders (25.9%) and PTSD (14.1%). Only one in three of the 465 current and ex-serving members who died by suicide between 2001 and 2018 were clients of DVA.^6^ Of the 146 males who were DVA clients at the time of their death, 75 had a processed claim for a mental health condition or conditions (47 for affective disorders; 38 for PTSD, acute stress reaction and adjustment disorders; 22 for psychoactive substance use; and 18 for neurotic, stress-related and somatoform disorders).

In the most recent AIHW (2022) report on suicide in current and ex-serving ADF members, 48.5% of males and 66.1% of females who died had been diagnosed with an affective disorder, slightly higher than in community controls (making it the most common group of mental illnesses associated with ADF suicide). In males who were discharged medically, this increased to 58.9% and was closely followed by anxiety and stress-related disorders, with a prevalence of 49.3%. For ADF females who died by suicide, 29.5% had been diagnosed with anxiety and stress-related disorders, similar to the rate for females who died by suicide in the general community (31.1%).^7^ A strong relationship between mental illness and suicidality was similarly found in the MHPW study (McFarlane et al., 2011). The vast majority of people (90%) who had attempted suicide met criteria for a mental disorder within the previous 12 months.

In a recent study of ageing Vietnam veterans, severe depression was again the strongest predictor of suicide ideation and planning, although there was also a strong independent association with PTSD (O’Toole et al., 2015). Alcohol dependence had the strongest association with suicide attempts. An earlier case-control study of overall mortality in Vietnam veterans (without specifically examining suicide) revealed psychiatric conditions and discharge with ‘abnormal emotional stability’ (as indicated by PULHEEMS coding of the discharge medical)^8^ were strongly associated with mortality (O’Toole et al., 1988). The study was later extended to examine suicide, there being 91 suicides among 523 total deaths in Vietnam and Vietnam-era veterans up to 1982 (O’Toole and Cantor, 1995). Again, psychiatric disorder and abnormal emotional stability on discharge were strongly associated with suicide.

### Health service utilisation

Health service utilisation statistics can be difficult to interpret due to overlapping entitlements to Defence-funded services, DVA-funded services and Medicare-subsidised services across the career and lifespan of serving and ex-serving members. With that in mind, the AIHW (2021) report finds that most ex-serving males (88%) and reserve males (85%) who died by suicide utilised at least one Medicare-subsidised or DVA-funded health service in the 12 months prior to their death. This is similar to the proportion of Australian males who accessed Medicare-subsidised services in the 12 months before their death by suicide (85%). Over half (53%) of ex-serving males who died by suicide accessed a mental health–related service in the 12 months prior to death, compared with 38% of age-matched males in the community. Almost one quarter (23%) of serving ADF members accessed DVA-funded or Medicare-subsidised services in the year before their death, despite the primary source of health care for serving members being Defence-funded.

### Transition and psychosocial risk factors

Psychosocial factors such as relationships, finance, accommodation and employment status have all been shown to be relevant to risk of suicide and suicidality (for example, Nock et al., 2013, a US study). The sudden change in employment status associated with transition out of the military (which in itself is often associated with other psychosocial stressors) appears to be particularly relevant (Kapur et al., 2009, a UK study; Reger et al., 2015, a US study). The period of time around transition out of the permanent forces, especially if for involuntary medical reasons, is a time of heightened risk (Commonwealth of Australia, 2021). The TWRP (Van Hooff et al., 2018) assessed suicidality in a cohort which transitioned between 2010 and 2014. The greatest differences in suicidality were between the group that was medically discharged and those who were discharged for other reasons. The 12-month prevalence of any suicidality in those who were discharged for medical reasons was 42.6%, compared with 16% in those who were discharged for other reasons. The TWRP also found that among those who had transitioned by 2015, 12.4% had reported any suicidality in 2010 and that this had increased to 27.4% in 2015, a proportionately much higher increase than in those who continued to serve in the regular ADF (Bryant et al., 2019).

The most recent AIHW (2022) suicide monitoring report found a suicide rate of 69.8/100,000/year in those male members discharged medically, compared with 22.5/100,000/year in those who discharged voluntarily.^9^ The most common psychosocial correlates for both males and females were problems in spousal relationship circumstances and problems related to employment and unemployment.

Kerr et al. (2018) performed a retrospective cohort study of 229 ex-serving personnel attending a PTSD day programme at a private hospital in Brisbane. Past history of suicide attempt in this cohort was significantly associated with being unemployed and/or totally and permanently incapacitated and PTSD symptom severity.

### Impact of deployment

The Third (and most recent) Australian Vietnam Veterans Mortality Study (Wilson et al., 2005) reported on suicide in the entire cohort of personnel who deployed to Vietnam, and compared this to the matched Australian community. A total of 421 suicides were reported up until the end of 2001 (the limit of the study), with an SMR of 1.03. When analysed separately across approximately each decade since the end of the war, however, there was a trend of increasing SMR for suicide with time (from 0.86 to 1.15).

The 2010 ADF MHPW study (McFarlane et al., 2011) examined the impact on suicidality of having ever deployed. There was no significant difference in prevalence of any suicidality between those who had ever deployed and those who had never deployed. Only the prevalence of thoughts that ‘life was not worth living’ showed a small but statistically significant difference, with deployment being protective. The study likewise found very little difference in prevalence of mental disorders between those who had deployed and those who had not.

Nested within the TWRP (Van Hooff et al., 2018) was an Impact of Combat Study (Lawrence-Wood et al., 2019) which examined inter alia changes in mental well-being in a cohort of ADF members deployed to the Middle East Area of Operations between 2010 and 2012. Data were collected pre-deployment, early post-deployment and in the 2015 TWRP data collection. A total of 560 veterans completed all three phases of data collection, 129 of whom had transitioned out of the ADF by the third and final time point. While there was a small increase in any suicidality between the pre-deployment and post-deployment measures, there was a large increase at the third (2.2%, 3.6% and 12.7%, respectively). There had been no reports of suicide planning or attempted suicide in the cohort until the third time point (Lawrence-Wood et al., 2019). While the greater TWRP study cohort had a significant difference in suicidality between transitioned and still serving personnel, but not in the 12-month prevalence of suicidality between those who had ever deployed or never deployed in the transitioned cohort (see above), the Impact of Combat Study did not report on the difference in suicidality between the transitioned and still serving cohort. The study did, nevertheless, demonstrate significantly higher prevalence of mental disorders among the transitioned members of the deployed cohort (Lawrence-Wood et al., 2019).

The Australian Gulf War Veterans’ Follow Up Health Study examined suicidality in the cohort of Australian servicemen and women who deployed to the Arabian Gulf region for the 1991 Gulf War (Sim et al., 2015). Compared with a group of members serving in operational units at the time of the Gulf War, but not deployed, Gulf War veterans were significantly more likely to feel that life was not worth living and of making a suicide plan. There was no difference in attempted suicide or suicide mortality.

Forbes et al. (2016) investigated suicidality in a randomly selected cohort of Australian veterans of peacekeeping missions between 1989 and 2002. The comparison population was again drawn from the 2007 National Survey of Mental Health and Wellbeing (Australian Bureau of Statistics, 2008). The 12-month prevalence of suicide ideation, planning and attempt, at 10.7%, 5.8% and 1.0%, respectively, was all significantly higher than in the comparator sample (2.7%, 0.7% and 0.2%, respectively). While the relationship between suicidality and potentially traumatic events on deployment was not assessed, Forbes et al. (2016) did find a relationship between the latter and prevalence of PTSD in the peacekeepers.

### Other associations

Data from the TWRP were utilised to examine the association between ‘problem anger’ and suicidality in current- and ex-serving personnel (Varker et al., 2022). Anger was associated with diagnoses of depression, PTSD and alcohol dependence, although an association between anger and suicidality was significant even when controlling for mental health problems. Anger was also noted to increase significantly after separation from the ADF.

Data from the ADF MHPW study and the TWRP were used to assess the role of childhood determinants of military suicidality. Childhood anxiety was found to be strongly and independently associated with suicidality in the serving ADF (Syed Sheriff et al., 2019), while suicidality in recently transitioned men was associated with childhood-onset interpersonal trauma and anxiety (Syed Sheriff et al., 2020).

A study of ex-serving members attending a day programme for PTSD showed an association between experience of guilt and past suicide attempt (Kerr et al., 2021), and suicidality in Vietnam war veterans has also been shown to be significantly associated with psychotropic polypharmacy (Theal et al., 2020). A small qualitative study has described the potential reduction in suicidal ideation and suicide in Australian veterans with PTSD due to the influence of a service/assistance dog (McLaughlin and Hamilton, 2019). Distraction from suicidal thoughts and a sense of responsibility to the dog were suggested as being protective.

## Discussion

To our knowledge, this is the first comprehensive scoping review of the literature concerning suicide and suicidality in the Australian veteran population. This has permitted a synthesis of all existing peer-reviewed and grey literature in the area. As discussed below, there is emerging precision around the scale of problem of suicide death among veterans, although there is still much to understand about veteran suicidality. This is despite suicidality being a significant cause of distress and a phenomenon which in most cases precedes suicide death – thereby presenting a key opportunity for intervention.

The findings reported here span almost 50 years of research and should therefore also be considered as reflective of the era in which they were conducted and when the individuals rendered their service. Approaches to recruitment, training and transition, the nature of mental health support services and community attitudes towards mental illness (both in the military and more broadly) have all evolved over this period, such that the most recent research will have most weight in formulating implications and directing future research.

### Suicide

It is only since 2017 that rigorous epidemiological studies, conducted outside Defence but linking Defence personnel data with national mortality data, have been able to provide a reasonably accurate picture of veteran suicide. The size of the veteran cohort under surveillance has been substantially expanded since 2017 with Defence’s contribution of data from legacy personnel administration systems. As a consequence, a number of important and reliable observations have emerged. Age-adjusted suicide in serving males is approximately half that of community levels (AIHW, 2022). This may be, in part, explained by the so-called ‘healthy worker’ or ‘healthy soldier’ effect (Baillargeon, 2001; McLaughlin et al., 2008), owing to the screened nature of the population, relative good health and physical fitness, and the psychosocial benefits of being in stable employment, not least of all the military sense of belonging (see, for example, Joiner, 2005). Figures for serving females appear to show similar trends but are based on low absolute numbers. It is in the ex-serving cohort that the suicide incidence climbs significantly, especially so with involuntary medical discharge.

The presence of mental illness is important (AIHW, 2021, 2022), severe presentations of which are likely over-represented in the medically discharged group. It also provides an important target for intervention. Affective disorders, alcohol use disorders and PTSD are identified as particularly prevalent among those who have died by suicide. There is a great deal of health service utilisation in the lead-up to suicide, but it is fragmented across a complex variety of service systems which serving and ex-serving Defence members have access to (AIHW, 2021). Strikingly, only one-third of those who died by suicide were DVA clients at the time of their death and, of these, approximately half had a processed claim for a mental health condition (AIHW, 2021). It is acknowledged, however, that significant efforts have been made since the release of those statistics to improve the handover from Defence to DVA for those who are transitioning out of Defence (Commonwealth of Australia, 2022). The Australian Government has also agreed to relevant recommendations set out in the Interim Report of the Royal Commission, such as simplifying veteran compensation and rehabilitation legislation, clearing a large backlog of claims and improving the administration of the claims system (Australian Government, 2022).

### Suicidality

Perhaps the most significant, and certainly the most comprehensive, studies of suicidality in the ADF and soon after transition were the MHPW (McFarlane et al., 2011) and TWRP (Van Hooff et al., 2018) studies. The MHPW study reported suicidal ideation and planning at double the rate in the community. The suicide attempt rate was similar to the community, and from the linkage studies of the AIHW we know that the suicide rate in permanent serving members is half that of the community. Thus, we see an inverted pattern of increased suicidality and reduced suicide in serving members. There could be a number of reasons why the increased level of suicidality does not translate into higher levels of suicide, such as high levels of education and training in mental health awareness, emphasis on the ‘buddy’ system and looking after your mates, a well-developed welfare system with multiple points of access to care and ready access to primary health care. The extent to which any or all of these confer protection is unknown.

The TWRP study’s longitudinal design demonstrated a more than doubling of 12-month suicidality in current serving permanent members between 2010 and 2015 (up from 4.0% to 8.8%). This was associated with smaller increases in psychological distress, PTSD, depression and anger, the authors suggesting that the findings could be related to the high operational tempo at the time (Van Hooff et al., 2019). Other possible explanations might include improved case ascertainment and greater preparedness to report associated with education, training and efforts to reduce stigma. What is very clear from the TWRP study, though, is that those members who had transitioned out of the ADF had markedly increased rates of suicidal ideation, planning and attempts, including in the years prior to transition (Bryant et al., 2019). Suicidality was significantly higher still in those who were discharged for medical reasons (Van Hooff et al., 2018). Of course, the medically transitioned cohort will include a proportion of people with mental illness and psychological distress related to other factors such as chronic physical pain and substance use, but even those who have discharged for non-medical reasons have markedly increased rates of suicidality. Increased suicide and/or suicidality post-transition have been reported in other militaries, or specific cohorts within them, as well (Brignone et al., 2017; Kapur et al., 2009; Ravindran et al., 2020; Reger et al., 2015).

Australian research has identified a variety of psychosocial risk factors for suicide and suicidality. Difficulties in spousal relationships and post-separation employment have been identified as risk factors (AIHW, 2022). Experiences of anger (Varker et al., 2022) and guilt (Kerr et al., 2021) can be risk factors. Factors which precede service, such as childhood trauma and anxiety, may also contribute to risk of suicidality (Syed Sheriff et al., 2019, 2020).

### Deployment

The Australian Vietnam Veterans Mortality Study (Wilson et al., 2005) was the first large-scale study to compare Vietnam veteran suicide with matched community data. They detected a trend of increasing suicide risk over time; this remains a concern as many Vietnam veterans are still alive today, and more recent studies of this ageing cohort detect alarmingly high levels of suicidality (O’Toole et al., 2015).

The Australian Gulf War Veterans’ Follow Up Health Study (Sim et al., 2015) investigated suicidality rather than suicide, and it detected significantly elevated levels of suicidal ideation, but not suicide attempts, in the deployed cohort versus a matched non-deployed cohort. The authors linked the higher suicidality in the deployed cohort to elevated levels of demoralisation (Kissane et al., 2004); however, this does not explain the absence of a difference in suicide attempts between the two groups.

A study of peacekeepers deployed between 1989 and 2002 found significantly elevated levels of suicidality compared to a matched community comparator (Forbes et al., 2016). Importantly, Forbes et al. (2016) also detected a relationship between potentially traumatic events on deployment and the development of PTSD, highlighting that the specific experiences of deployment, more so than deployment per se, need to be considered.

The MHPW study (McFarlane et al., 2011) did not detect increased suicidality in personnel who had ever deployed compared with those who had never deployed, and the deployment component of the TWRP study (Lawrence-Wood et al., 2019), which examined a cohort of contemporary veterans from the Middle East, detected what appears to be a stronger impact of having transitioned out of the ADF than the deployment experience itself.

The importance of considering the nature of the deployment and the nature of specific traumatic exposures on that deployment (not only engagement in combat but also exposures such as threat of violence, witnessing suffering, handling human remains and non-combat-related accidents, for example) is illustrated by the above findings and has also been highlighted in studies of US veterans (Bryan et al., 2015). Consideration should also be given to associated factors around the time of increased operational tempo, such as the nature of recruitment, training, reception on return to Australia and community attitudes towards specific deployments.

### Strengths and limitations

To our knowledge, this is the first formal scoping review of suicide and suicidality in Australian veterans. Multiple databases spanning the medical, nursing, allied health and biomedical literature were utilised. Grey literature was considered, including several large and important studies which were published as reports. While the language of manuscripts was limited to English, this was not considered a limiting factor for Australian veteran material. Overall, we are confident that important studies, reports and manuscripts have not been missed. Utilising a scoping review methodology, which is intended to be inclusive of the broad literature, there was no risk of bias assessment of the studies and reports (Tricco et al., 2018).

A major limiting factor in much of suicide and suicidality research is the variability in definitions, analytical approach and case ascertainment, although recent large linkage studies have achieved significant improvements in the quality of veteran suicide research specifically (for example, AIHW, 2022). Suicidality research continues to be beset with these limitations.

## Conclusion

There has long been a popular impression that military service is associated with trauma, psychological distress, suicidality and suicide; the ongoing Royal Commission into Defence and Veteran Suicide has brought some of these matters into wider awareness and, importantly, is an opportunity to examine the evidence base and identify important areas for further study. It is clear that the risk of suicide and suicidality in veterans cannot be understood reductively in relation solely to mental illness or operational deployment.

Australian research to date has identified that during the period that a person is serving, service is associated with increased suicidality, but that it may be protective against suicide mortality. The specific features of service which contribute to suicidality during this period, but protect from suicide death, are not known and worthy of further attention. It is noteworthy, however, that the contemporary AIHW comparisons of suicide mortality in current serving members are made with the general Australian population matched for age and sex (AIHW, 2022), but not employment status, and any analysis matched for employment may diminish, partially or entirely, any perceived protective effect of service above the fact of employment (Skinner et al., 2023).

Transition from service is associated with increased risk of both suicidality and suicide deaths. There is a consistent and significant overrepresentation of suicide and suicidality in those veterans who have transitioned. Investigators have examined many factors for association with suicide and suicidality (factors such as age, gender, rank, years of service, nature of separation, deployment history, mental illness and so on); however, important unifying themes for all military separation can be found in the interpersonal theory of suicide: those of perceived burdensomeness and thwarted belongingness (Joiner, 2005). These factors have been the basis for many studies in the US military setting (for example, Chu et al., 2017; Hom et al., 2019). The extent to which such factors, and also psychosocial factors such as relationships and post-transition finances and employment, and pre-service factors, are causal or mediating, is worthy of further exploration.

There are some other potential associations with suicide and suicidality which have little or no coverage in the literature reviewed here, and are worthy of further attention. One emerging area is moral injury, the concept that psychosocial harm can follow events which transgress one’s deeply held moral convictions or value systems (Phelps et al., 2024). Another is the impact of historical and contemporary abuse and bullying on veterans (Livingston et al., 2022). From a medical point of view, further understanding of the interplay with co-morbidities such as physical injury, particularly including head injury (Howlett et al., 2022), chronic pain (Blakey et al., 2018) and substance use (Na et al., 2021), may help direct prevention and treatment efforts.

Transition and mental illness are important, and may be more important than a history of deployment (notwithstanding specific traumatic exposures that are relevant in a subset of those who deploy). We have a poor appreciation, however, of where and how transitioning members access health care. Peri- and post-transition, veterans can choose to access care through Defence internally (which may involve providers internal or external to Defence), privately through Medicare Benefits Schedule-rebateable or non-rebateable services, state-operated hospital and crisis response services or through DVA-funded services. A better understanding of how veterans enter, move through and benefit from these varied and often poorly coordinated services, particularly when in crisis, is likely to assist in directing resources more effectively and efficiently.

## Supplemental Material

sj-docx-1-anp-10.1177_00048674241246443 – Supplemental material for Suicide and suicidality in Australian Defence Force veterans: A systematic scoping review Supplemental material, sj-docx-1-anp-10.1177_00048674241246443 for Suicide and suicidality in Australian Defence Force veterans: A systematic scoping review by Csongor G Oltvolgyi, Carla Meurk and Ed Heffernan in Australian & New Zealand Journal of Psychiatry

## Notes

1. Specifically, coronial data are uploaded to the National Coronial Information System (NCIS) and then assessed and coded for cause of death by the Australian Bureau of Statistics (ABS), before being entered onto the National Mortality Database (NMD) held by the Australian Institute of Health and Welfare (AIHW).

2. It is acknowledged that the statistics under discussion here represent individual human lives lost and immeasurable pain for countless others touched by these deaths. It is hoped that scientific discussion contributes positively to prevention efforts.

3. Statistics regarding female suicide is to be interpreted with caution due to low overall numbers of female suicide deaths.

4. The 2023 update to the AIHW annual report (AIHW, 2023) was published after submission of this manuscript for review and therefore outside its search parameters. The 2023 report provides similar data with an additional year of reporting (up to 2021) and takes account of any revisions to coronial data. The total number of suicides is revised to 1677, 1542 in males and 1395 in the ex-serving population. The age-adjusted comparisons to Australian community figures are unchanged except that the figures for males in the reserves are 45% lower, not 46%. The suicide rate in males subject to involuntary medical separation is revised to 67.1/100,000/year. Other relevant updates are included as brief footnotes, below.

5. Many researchers would argue that non-suicidal self-injury (NSSI) is a phenomenon distinct from suicidality in any case (Turecki et al., 2019), although it is often considered itself a risk factor for suicide (Grandclerc et al., 2016).

6. The latest AIHW (2023) report updates the proportion of ex-serving males and females who were Department of Veterans’ Affairs (DVA) clients at the time of suicide as 29.1% and 24.8%, respectively (ascertained from 2002 to 2021). Among males who were still serving at the time of suicide, 41% were DVA clients. The difference in suicide rates between those who were and who were not DVA clients at the time of their deaths was not statistically significant.

7. The mental illness correlates of veteran suicide were not updated and reported in the latest AIHW (2023) report.

8. PULHEEMS is a coding system used by the Australian Army for medical fitness where P = physical capacity, U = upper limbs, L = lower limbs, H = hearing, EE = each eye, M = mental capacity and S = emotional stability.

9. The latest AIHW (2023) report amends these figures slightly to 67.1/100,000/year and 21.5/100,000/year, respectively.

## References

[bibr1-00048674241246443] Adena MA Cobbin DM Fett MJ , et al. (1985) Mortality among Vietnam veterans compared with non-veterans and the Australian population. Medical Journal of Australia 143: 541–544. 3831743 10.5694/j.1326-5377.1985.tb119945.x

[bibr2-00048674241246443] AIHW (2021) Final Report to the Independent Review of past Defence and Veteran Suicides. PHE 295, 2021. Canberra, ACT, Australia: AIHW.

[bibr3-00048674241246443] AIHW (2022) Serving and Ex-Serving Australian Defence Force Members Who Have Served since 1985: Suicide Monitoring 1997 to 2020. PHE 315, 2022. Canberra, ACT, Australia: AIHW.

[bibr4-00048674241246443] AIHW (2023) Serving and Ex-Serving Australian Defence Force Members Who Have Served since 1985: Suicide Monitoring 1997 to 2021. PHE 327, 2023. Canberra, ACT, Australia: AIHW.

[bibr5-00048674241246443] Australian Bureau of Statistics (2008) National Survey of Mental Health and Wellbeing 2007. Canberra, ACT, Australia: Australian Bureau of Statistics.

[bibr6-00048674241246443] Australian Bureau of Statistics (2021) Service with the Australian Defence Force: Census 2021. Canberra, ACT, Australia: Australian Bureau of Statistics.

[bibr7-00048674241246443] Australian Government (2022) Australian Government Response to the Interim Report of the Royal Commission into Defence and Veteran Suicide. Canberra, ACT, Australia: Department of Defence and Department of Veterans’ Affairs.

[bibr8-00048674241246443] Baillargeon J (2001) Characteristics of the healthy worker effect. Occupational Medicine 16: 359–366. 11319057

[bibr9-00048674241246443] Bertolote J Wasserman D (2021) Development of definitions of suicidal behaviours: From suicidal thoughts to completed suicides. In: Wasserman D (ed.) Oxford Textbook of Suicidology and Suicide Prevention, 2nd Edition. Oxford: Oxford University Press, pp. 87–90.

[bibr10-00048674241246443] Biddle N Ellen L Korda R , et al. (2020) Suicide Mortality in Australia: Estimating and Projecting Monthly Variation and Trends from 2007 to 2018 and beyond. Canberra, ACT, Australia: Australian National University.

[bibr11-00048674241246443] Blakey SM Wagner HR Naylor J , et al. (2018) Chronic pain, TBI, and PTSD in military veterans: A link to suicidal ideation and violent impulses? The Journal of Pain 19: 797–806. 29526669 10.1016/j.jpain.2018.02.012 PMC6026045

[bibr12-00048674241246443] Boman B (1985) Post-traumatic stress disorder (traumatic war neurosis) and concurrent psychiatric illness among Australian Vietnam veterans. A controlled study. Journal of the Royal Army Medical Corps 131: 128–131. 4087237 10.1136/jramc-131-03-02

[bibr13-00048674241246443] Brignone E Fargo JD Blais RK , et al. (2017) Non-routine discharge from military service: Mental illness, substance use disorders, and suicidality. American Journal of Preventive Medicine 52: 557–565. 28109642 10.1016/j.amepre.2016.11.015

[bibr14-00048674241246443] Bryan CJ Griffith JE Pace BT , et al. (2015) Combat exposure and risk for suicidal thoughts and behaviors among military personnel and veterans: A systematic review and meta-analysis. Suicide & Life-Threatening Behavior 45: 633–649. 29889337 10.1111/sltb.12163

[bibr15-00048674241246443] Bryant RA (1998) An analysis of calls to a Vietnam veterans’ telephone counselling service. Journal of Traumatic Stress 11: 589–596. 9690196 10.1023/A:1024417031977

[bibr16-00048674241246443] Bryant R Lawrence-Wood E Baur J , et al. (2019) Mental Health Changes Over Time: A Longitudinal Perspective (Mental Health and Wellbeing Transition Study). Canberra, ACT, Australia: Department of Defence and Department of Veterans’ Affairs.

[bibr17-00048674241246443] Chu C Buchman-Schmitt JM Stanley IH , et al. (2017) The interpersonal theory of suicide: A systematic review and meta-analysis of a decade of cross-national research. Psychological Bulletin 143: 1313–1345. 29072480 10.1037/bul0000123 PMC5730496

[bibr18-00048674241246443] Commonwealth of Australia (2021) Preliminary Interim Report: Interim National Commissioner for Defence and Veteran Suicide Prevention. Canberra, ACT, Australia: Commonwealth of Australia.

[bibr19-00048674241246443] Commonwealth of Australia (2022) Royal Commission into Defence and Veteran Suicide: Interim Report. Canberra, ACT, Australia: Commonwealth of Australia.

[bibr20-00048674241246443] Duncan DR O’Gorman JG Fleming KJ , et al. (1974) Suicidal behaviour in the Australian army: Incidence, methods, and outcome. Medical Journal of Australia 2: 736–739. 4444629 10.5694/j.1326-5377.1974.tb71129.x

[bibr21-00048674241246443] Favril L Yu R Geddes JR , et al. (2023) Individual-level risk factors for suicide mortality in the general population: An umbrella review. Lancet Public Health 8: e868–e877. 10.1016/S2468-2667(23)00207-4 PMC10932753 37898519

[bibr22-00048674241246443] Fett MJ Adena MA Cobbin DM , et al. (1987a) Mortality among Australian conscripts of the Vietnam conflict era. I. Death from all causes. American Journal of Epidemiology 125: 869–877. 3565361 10.1093/oxfordjournals.aje.a114603

[bibr23-00048674241246443] Fett MJ Nairn JR Cobbin DM , et al. (1987b) Mortality among Australian conscripts of the Vietnam conflict era. II. Causes of death. American Journal of Epidemiology 125: 878–884. 3565362 10.1093/oxfordjournals.aje.a114604

[bibr24-00048674241246443] Forbes D O’Donnell M Brand RM , et al. (2016) The long-term mental health impact of peacekeeping: Prevalence and predictors of psychiatric disorder. BJPsych Open 2: 32–37. 27703751 10.1192/bjpo.bp.115.001321 PMC4995565

[bibr25-00048674241246443] Forcier L Hudson HM Cobbin DM , et al. (1987) Mortality of Australian veterans of the Vietnam Conflict and the period and location of their Vietnam service. Military Medicine 152: 117–124. 3104823

[bibr26-00048674241246443] Gisler K Sadler N (2000) Suicide in the ADF (1985-2000). Australian Military Medicine 9: 138–142.

[bibr27-00048674241246443] Grandclerc S De Labrouhe D Spondenkiewicz M , et al. (2016) Relations between nonsuicidal self-injury and suicidal behaviour in adolescence: A systematic review. PLoS ONE 11: e0153760. 10.1371/journal.pone.0153760 PMC4835048 27089157

[bibr28-00048674241246443] Harris EC Barraclough B (1997) Suicide as an outcome for mental disorders. A meta-analysis. The British Journal of Psychiatry 170: 205–228. 9229027 10.1192/bjp.170.3.205

[bibr29-00048674241246443] Hom MA Duffy ME Rogers ML , et al. (2019) Examining the link between prior suicidality and subsequent suicidal ideation among high-risk US military service members. Psychological Medicine 49: 2237–2246. 30355371 10.1017/S0033291718003124

[bibr30-00048674241246443] Howlett JR Nelson LD Stein MB (2022) Mental health consequences of traumatic brain injury. Biological Psychiatry 91: 413–420. 34893317 10.1016/j.biopsych.2021.09.024 PMC8849136

[bibr31-00048674241246443] Joiner TE (2005) Why People Die by Suicide. Cambridge, MA: Harvard University Press.

[bibr32-00048674241246443] Jowett S Carpenter B Tait G (2019) Determining a suicide under Australian law: A comparative study of coronial practice. University of New South Wales Law Journal 42: 534–556.

[bibr33-00048674241246443] Kapur N Goldney RD (2019) Suicide Prevention. Oxford: Oxford University Press.

[bibr34-00048674241246443] Kapur N While D Blatchley N , et al. (2009) Suicide after leaving the UK armed forces: A cohort study. PLoS Medicine 6: 0269–0277. 10.1371/journal.pmed.1000026 PMC2650723 19260757

[bibr35-00048674241246443] Kerr K Romaniuk M McLeay S , et al. (2018) Increased risk of attempted suicide in Australian veterans is associated with total and permanent incapacitation, unemployment and posttraumatic stress disorder severity. Australian and New Zealand Journal of Psychiatry 52: 552–560. 28707521 10.1177/0004867417718945

[bibr36-00048674241246443] Kerr K Romaniuk M McLeay S , et al. (2021) Guilt and its relationship to mental illness and suicide attempts in an Australian veteran population with posttraumatic stress disorder. Journal of Military and Veterans’ Health 29: 7–15.

[bibr37-00048674241246443] Kerridge I Lowe M Stewart C (2013) Ethics and Law for the Health Professions. Sydney, NSW, Australia: The Federation Press.

[bibr38-00048674241246443] Kissane DW Wein S Love A , et al. (2004) The demoralization scale: A report of its development and preliminary validation. Journal of Palliative Care 20: 269–276. 15690829

[bibr39-00048674241246443] Klonsky ED May AM (2015) The three-step theory (3ST): A new theory of suicide rooted in the ‘ideation-to-action’ framework. International Journal of Cognitive Therapy 8: 114–129.

[bibr40-00048674241246443] Lane J Wallace D (2020) Australian military and veteran’s mental health care part 1: An introduction to cultural essentials for clinicians. Australasian Psychiatry 28: 267–269. 32019355 10.1177/1039856220901470

[bibr41-00048674241246443] Lawrence-Wood E McFarlane A Lawrence A , et al. (2019) Impact of Combat Report (Impact of Combat Study). Canberra, ACT, Australia: Department of Defence and Department of Veterans’ Affairs.

[bibr42-00048674241246443] Livingston WS Tannahill HS Meter DJ , et al. (2022) The association of military sexual harassment/assault with suicide ideation, plans, attempts, and mortality among US service members/veterans: A meta-analysis. Trauma, Violence & Abuse 24: 2616–2629. 10.1177/15248380221109790 35763372

[bibr43-00048674241246443] McFarlane A Hodson S Van Hoof M , et al. (2011) Mental Health in the Australian Defence Force: 2010 ADF Mental Health Prevalence and Wellbeing Study: Full Report. Canberra, ACT, Australia: Department of Defence.

[bibr44-00048674241246443] McLaughlin K Hamilton AL (2019) Exploring the influence of service dogs on participation in daily occupations by veterans with PTSD: A pilot study. Australian Occupational Therapy Journal 66: 648–655. 31512257 10.1111/1440-1630.12606

[bibr45-00048674241246443] McLaughlin R Nielsen L Waller M (2008) An evaluation of the effect of military service on mortality: Quantifying the healthy soldier effect. Annals of Epidemiology 18: 928–936. 19041592 10.1016/j.annepidem.2008.09.002

[bibr46-00048674241246443] Milner A Witt K Maheen H , et al. (2017) Suicide among emergency and protective service workers: A retrospective mortality study in Australia, 2001 to 2012. Work 57: 281–287. 28582946 10.3233/WOR-172554

[bibr47-00048674241246443] Munn Z Peters MDJ Stern C , et al. (2018) Systematic review or scoping review? Guidance for authors when choosing between a systematic or scoping review approach. BMC Medical Research Methodology 18: 143–143. 30453902 10.1186/s12874-018-0611-x PMC6245623

[bibr48-00048674241246443] Na PJ Nichter B Hill ML , et al. (2021) Severity of substance use as an indicator of suicide risk among U.S. military veterans. Addictive Behaviors 122: 107035. 34246987 10.1016/j.addbeh.2021.107035

[bibr49-00048674241246443] Nock MK Deming CA Fullerton CS , et al. (2013) Suicide among soldiers: A review of psychosocial risk and protective factors. Psychiatry 76: 97–125. 23631542 10.1521/psyc.2013.76.2.97 PMC4060831

[bibr50-00048674241246443] O’Connor RC (2011) The integrated motivational-volitional model of suicidal behavior. Crisis 32: 295–298. 21945841 10.1027/0227-5910/a000120

[bibr51-00048674241246443] O’Toole BI Adena MA Jones MP (1988) Risk factors for mortality in Australian Vietnam-era national servicemen: A case-control study. Community Health Studies 12: 408–417. 3243075 10.1111/j.1753-6405.1988.tb00607.x

[bibr52-00048674241246443] O’Toole BI Cantor C (1995) Suicide risk factors among Australian Vietnam era draftees. Suicide & Life-Threatening Behavior 25: 475–488. 8928202

[bibr53-00048674241246443] O’Toole BI Catts SV Outram S , et al. (2010) Factors associated with civilian mortality in Australian Vietnam veterans three decades after the war. Military Medicine 175: 88–95. 20180477 10.7205/milmed-d-09-00071

[bibr54-00048674241246443] O’Toole BI Orreal-Scarborough T Johnston D , et al. (2015) Suicidality in Australian Vietnam veterans and their partners. Journal of Psychiatric Research 65: 30–36. 25914085 10.1016/j.jpsychires.2015.02.003

[bibr55-00048674241246443] Page MJ McKenzie JE Bossuyt PM , et al. (2021) The PRISMA 2020 statement: An updated guideline for reporting systematic reviews. British Medical Journal 372: n71. 10.1136/bmj.n71 PMC8005924 33782057

[bibr56-00048674241246443] Peters MDJ Marnie C Tricco AC , et al. (2020) Updated methodological guidance for the conduct of scoping reviews. JBI Evidence Synthesis 18: 2119–2126. 33038124 10.11124/JBIES-20-00167

[bibr57-00048674241246443] Phelps AJ Adler AB Belanger SAH , et al. (2024) Addressing moral injury in the military. BMJ Military Health 170: 51–55. 35705259 10.1136/bmjmilitary-2022-002128

[bibr58-00048674241246443] Ravindran C Morley SW Stephens BM , et al. (2020) Association of suicide risk with transition to civilian life among US military service members. JAMA Network Open 3: e2016261. 10.1001/jamanetworkopen.2020.16261 PMC7489860 32915235

[bibr59-00048674241246443] Reger MA Smolenski DJ Skopp NA , et al. (2015) Risk of suicide among US military service members following Operation Enduring Freedom or Operation Iraqi Freedom deployment and separation from the US military. JAMA Psychiatry 72: 561–569. 25830941 10.1001/jamapsychiatry.2014.3195

[bibr60-00048674241246443] Sadler N Van Hooff M Bryant RA , et al. (2021) Suicide and suicidality in contemporary serving and ex-serving Australian Defence Force personnel. Australian and New Zealand Journal of Psychiatry 55: 463–475. 33726567 10.1177/0004867421998751

[bibr61-00048674241246443] Sim M Clarke D Forbes A , et al. (2015) Australian Gulf War Veterans’ Follow Up Health Study: Technical Report. Melbourne, VIC, Australia: Monash University.

[bibr62-00048674241246443] Skinner A Osgood ND Occhipinti J-A , et al. (2023) Unemployment and underemployment are causes of suicide. Science Advances 9: eadg3758. 10.1126/sciadv.adg3758 PMC10337900 37436996

[bibr63-00048674241246443] Syed Sheriff R Van Hooff M Malhi G , et al. (2019) Childhood determinants of suicidality: Comparing males in military and civilian employed populations. Psychological Medicine 49: 2421–2431. 30419985 10.1017/S0033291718003355

[bibr64-00048674241246443] Syed Sheriff R Van Hooff M Malhi GS , et al. (2020) Childhood determinants of suicidality in men recently transitioned from regular military service. Australian and New Zealand Journal of Psychiatry 54: 743–754. 32536196 10.1177/0004867420924742

[bibr65-00048674241246443] Theal R McLeay S Gibson J , et al. (2020) Psychotropic polypharmacy in Australian Vietnam war veterans with post-traumatic stress disorder: A descriptive cohort study. Journal of Military and Veterans’ Health 28: 34–45.

[bibr66-00048674241246443] Tricco AC Lillie E Zarin W , et al. (2018) PRISMA extension for scoping reviews (PRISMA-ScR): Checklist and explanation. Annals of Internal Medicine 169: 467–473. 30178033 10.7326/M18-0850

[bibr67-00048674241246443] Turecki G Brent DA Gunnell D , et al. (2019) Suicide and suicide risk. Nature Reviews Disease Primers 5: 74. 10.1038/s41572-019-0121-0 31649257

[bibr68-00048674241246443] Van Hooff M Lawrence-Wood E Hodson S , et al. (2018) Mental Health Prevalence, Mental Health and Wellbeing Transition Study. Canberra, ACT, Australia: Department of Defence and Department of Veterans’ Affairs.

[bibr69-00048674241246443] Van Hooff M Lawrence-Wood E Sadler N , et al. (2019) Transition and Wellbeing Research Programme Key Findings Report. Canberra, ACT, Australia: Department of Defence and Department of Veterans’ Affairs.

[bibr70-00048674241246443] Varker T Cowlishaw S Baur J , et al. (2022) Problem anger in veterans and military personnel: Prevalence, predictors, and associated harms of suicide and violence. Journal of Psychiatric Research 151: 57–64. 35453092 10.1016/j.jpsychires.2022.04.004

[bibr71-00048674241246443] Veritas Health Innovation (2023) Covidence. Melbourne, VIC, Australia: Veritas Health Innovation.

[bibr72-00048674241246443] Veterans’ Ministers Roundtable (2017) Joint Communique: Veterans’ Ministers Meeting 8 Nov 2017. https://www.aph.gov.au/About_Parliament/Parliamentary_Departments/Parliamentary_Library/FlagPost/2019/July/Recognition_of_Australian_Veterans

[bibr73-00048674241246443] Wilson E Horsley K van der Hoek R (2005) Australian Vietnam Veterans Mortality Study 2005. P1055b, 2005. Canberra, ACT, Australia: Department of Veterans’ Affairs.

