# Supplementary material for: Suicide and suicidality in Australian Defence Force veterans: A systematic scoping review
Source: Aust N Z J Psychiatry. 2024 Apr 22;58(9):760–74. doi: 10.1177/00048674241246443 (PMC11370192; doi:10.1177/00048674241246443)
Supplement: sj-docx-1-anp-10.1177_00048674241246443 – Supplemental material for Suicide and suicidality in Australian Defence Force veterans: A systematic scoping review [file sj-docx-1-anp-10.1177_00048674241246443.docx]

Suicide and suicidality in Australian Defence Force veterans:

A systematic scoping review

(Oltvolgyi, Meurk, Heffernan)

**Supplementary Materials 1**

**Search terms**

Searches conducted 23/01/23

PubMed/MEDLINE search terms:

| #19 | #11 AND #18 |
| --- | --- |
| #18 | #12 OR #13 OR #14 OR #15 OR #16 OR #17 |
| #17 | 'mortality'/exp OR mortality |
| #16 | 'self harm'/exp OR 'self harm' OR (('self'/exp OR self) AND harm) |
| #15 | 'automutilation'/exp OR automutilation |
| #14 | 'suicidality'/exp OR suicidality |
| #13 | 'suicidal behavior'/exp OR 'suicidal behavior' |
| #12 | 'suicide'/exp OR suicide |
| #11 | #9 AND #10 |
| #10 | 'australia'/exp OR australia |
| #9 | #1 OR #2 OR #3 OR #4 OR #5 OR #6 OR #7 OR #8 |
| #8 | 'defence force' OR (defence AND ('force'/exp OR force)) |
| #7 | 'armed forces'/exp OR 'armed forces' OR (armed AND forces) |
| #6 | 'air force'/exp OR 'air force' |
| #5 | 'navy'/exp OR navy |
| #4 | 'army'/exp OR army |
| #3 | 'military personnel'/exp OR 'military personnel' |
| #2 | 'military personnel'/exp OR 'military personnel' |
| #1 | 'veteran'/exp OR veteran |

CINAHL search terms:

S14 S9 AND S10 AND S13

S13 S1 OR S11 OR S12

S12 mortality

S11 self harm or self injury or deliberate self harm or self mutilation or self injurious behavior

S10 australia

S9 S2 OR S3 OR S4 OR S5 OR S6 OR S7 OR S8

S8 “air force”

S7 navy

S6 army

S5 defense

S4 defence

S3 military

S2 veteran*

S1 suicid*

Embase search terms:

18 10 and 17

17 11 or 12 or 13 or 14 or 15 or 16

16 mortality.mp. or exp mortality/

15 self harm.mp. or exp automutilation/

14 automutilation.mp. or exp automutilation/

13 suicidality.mp. or exp suicidal behavior/

12 suicidal behavior.mp. or exp suicidal behavior/

11 exp suicide/ or suicide attempt/ or suicidal ideation/ or suicide*.mp.

10 8 and 9

9 australia*.mp. or exp Australia/

8 1 or 2 or 3 or 4 or 5 or 6 or 7

7 defense.mp.

6 defence.mp.

5 air force.mp. or exp air force/

4 navy.mp. or exp navy/

3 army.mp. or exp army/

2 military personnel.mp. or exp military personnel/

1 veteran.mp. or veteran/
